# Health-Related Quality of Life Among Postpartum Women in Jordan: A Cross-Sectional Study

**DOI:** 10.3390/healthcare14111593

**Published:** 2026-06-05

**Authors:** Mais Alkhalili, Hadeel Bani-Said, Yamamah Alhmaid, Arwa M. Al-Dekah, Ensaf Almomani, Lama Hamadneh, Shifaa’ Al Qa’qa’, Khairat Battah, Dima Hamarsheh, Silvia D. Boyajian, Ayat Alakhras

**Affiliations:** 1 Faculty of Medicine, Al-Balqa Applied University, Al-Salt 19117, Jordan; y.alhmaid@bau.edu.jo (Y.A.); ensaf.momani@bau.edu.jo (E.A.); lama.hamadneh@bau.edu.jo (L.H.); shqaqa@bau.edu.jo (S.A.Q.); kh.battah@bau.edu.jo (K.B.); d.hamarsheh@bau.edu.jo (D.H.); silvia.boyajian@bau.edu.jo (S.D.B.); ayatfayez2003@gmail.com (A.A.); 2 Ministry of Health, Amman 11118, Jordan; hzbanisaid20@med.just.edu.jo; 3 Faculty of Science and Arts, Jordan University of Science and Technology, Irbid 22110, Jordan; amaldekah15@sci.just.edu.jo

**Keywords:** postpartum, quality of life, maternal health, Jordan, SF-36

## Abstract

**Objective:** This study aimed to assess health-related quality of life (HRQoL) among postpartum women in Jordan and identify the factors that may influence it. **Methodology:** This study employed a cross-sectional design and was conducted among women who had given birth within the last year, selected through cluster randomization from four primary healthcare centers in Amman. Household resource quality of life was measured on the validated 36-Item Short Form Survey (SF-36), which covers eight domains of health. Descriptive statistics were calculated. Independent *t*-tests and one-way ANOVA were used to compare mean HRQoL scores across categories of socio-demographic variables (e.g., marital status, income, employment, feeding type). Pearson correlation was used to examine the relationship between age (the only continuous variable) and HRQoL domains. Statistical significance was set at *p* < 0.05. **Results:** The physical functioning had the highest HRQoL (62.48 ± 25.19), and the lowest HRQoL (34.59 ± 37.46) was found in role limitations due to physical health and emotional problems (36.37 ± 40.90). Key socio-demographic factors were highly related to HRQoL. Better general health perceptions (*p* = 0.003) and emotional well-being (*p* = 0.005) were found to be correlated with higher income. The married women scored much higher in physical functioning (*p* = 0.015) and emotional well-being (*p* = 0.013) than divorced women. Infant feeding methods and employment status were also significantly associated with certain domains of HRQoL. **Conclusions:** Postpartum women in Jordan experience low HRQoL, particularly in the domains related to role limitations. Socio-demographic factors were found to be crucial, wherein marital status and income are specific aspects. This study strongly recommends immediate integration of an appropriate multidimensional support program in postpartum care as an intervention toward improving maternal well-being.

## 1. Introduction

The body goes through several physical, physiological, and psychological changes after childbirth [1]. Commonly, this period is said to last about six weeks [2,3]. The American College of Obstetricians and Gynecologists extends it to 12 weeks, labeling it the “fourth trimester” [4]. It has further been described as belonging to the acute (24 h), early (7 days), and late (6 weeks to 6 months) phases that spotlight its crucial impacts on maternal health [5]. Women are prone to different health problems and diseases in this phase. Postpartum depression and anxiety are psychiatric diseases that may occur during this phase; psychosis is very rare [6]. Others include postpartum hemorrhage and cardiomyopathy [7,8]. These diseases can be harmful to the mother’s health as well as the child’s health. To render optimal care and appropriate interventions, HRQoL assessment is imperative.

WHO defined quality of life (QoL) as ‘an individual’s perception of their position in life in the context of the culture and value systems in which they live and in relation to their goals, expectations, standards, and concerns’ [9]. Quality of life is a broad concept; however, HRQoL can be described as a subjective perception of one’s physical and mental well-being over time [10]. The HRQoL measure allows for an assessment of the self-reported health status of problems by individuals and interventions targeted at those specific problems at the individual, population, and policymaking levels [11]. However important it is, HRQoL has largely been understudied across low- and middle-income settings wherein unique socio-cultural factors plus healthcare systems interact with women’s postpartum experiences.

Globally, it has been established that postpartum depression, sleep disturbances, parity, unplanned pregnancy, and social support are major determinants of HRQoL [12]. The socioeconomic differences are also very crucial. Lower household income and education of women have been associated with worse scores in the general health and emotional well-being domains [13].

Emerging studies in the Middle East have reiterated that postpartum women are highly vulnerable to compromised HRQoL by cultural, social, and healthcare system factors. For instance, a cross-sectional study carried out in Madinah, Saudi Arabia, found that postpartum depression and sleep disturbance were independently associated with reductions in QoL related to all its domains [14]. Similarly, socioeconomic status, education, and obstetric complications have been reported from Iran as key predictors of lower postpartum HRQoL through the MAPP-QOL scale [15]. More than half the postpartum women in Jimma, Ethiopia, had “low” HRQoL; unplanned pregnancy, higher parity, partner’s low education, and adverse outcomes of pregnancy were the risk factors associated with it [16]. More than half of the women in the postpartum period were found to be suffering from low HRQoL in another study from Ethiopia, with income, parity, and presence of obstetric complications as major predictors [17]. Studies have reported a high prevalence of postpartum depression during the COVID-19 pandemic. However, comprehensive assessments regarding HRQoL are very limited [18].

In Jordan, studies mainly cover antenatal HRQoL and postpartum depression. There is a gap when it comes to a comprehensive analysis of postpartum HRQoL. Higher parity and lower perceived social support were significantly associated with QoL during pregnancy in a study of healthy pregnant women in North Jordan [19]. This is the result of a study from the COVID-19 era in Jordan, whereby postpartum depression among mothers who had COVID-19 during pregnancy or after delivery was very high—over 67%—where smoking, severity of symptoms, and hospitalization were primary risk factors. While this speaks to psychological distress, it does not fully speak to general HRQoL [20]. Qualitative studies of the life experience of women in high-parity pregnancy from North Jordan indicated that perceived QoL was negatively influenced by discomfort, inadequate antenatal care, and other related social problems [21]. Though appreciated, family and social support systems are irregular in reality, making women prone to psychological distress and functional limitations after delivery [22].

To guide maternal health policy, design culturally responsive interventions, and direct healthcare efforts beyond survival rates to women’s holistic well-being, an understanding of postpartum HRQoL in Jordan is crucial. In the process, this study falls right within the scheme of things for WHO’s drive toward a good postnatal experience by generating evidence that would be very relevant directly for application in the Middle Eastern and Jordanian contexts.

The current study is the first cross-sectional estimation of the HRQoL in the conditions of postpartum women in Jordan based on the SF-36, a validated multidimensional instrument aimed at measuring HRQoL and factors affecting it in the target group. When a sample of women attending selected primary healthcare facilities in Amman was recruited, this study not only measured postpartum HRQoL but also explained the relationships between it and marital status, income, employment, delivery mode, and infant feeding behaviors.

## 2. Materials and Methods

### 2.1. Study Design and Setting

A cross-sectional study was conducted in government primary comprehensive healthcare centers in Amman, Jordan, from January to March 2025.

Sampling—Stage 1 (Cluster Random Sampling of Facilities): According to the Jordanian Ministry of Health, there are 121 primary comprehensive healthcare centers across Jordan, of which 24 are located in Amman [23]. These 24 centers were first stratified by geographical location into two clusters: West Amman (16 centers) and East Amman (8 centers). Using a random number table, two centers were randomly selected from West Amman (Sweileh and Al-Bayader comprehensive healthcare centers) and two from East Amman (Sahab and Al-Mqabalein comprehensive healthcare centers). This cluster randomization approach was chosen to ensure geographic representation of both socioeconomically diverse areas of Amman and to minimize selection bias at the facility level.

### 2.2. Study Population

Women aged 18 years or older who were less than or equal to 12 months postpartum at the time of interview and attending well-baby, immunization, family planning, or postnatal clinics at participating centers were included. Exclusion criteria were a major depressive episode at present; serious chronic conditions that may prevent an individual from performing daily tasks (e.g., advanced cardiac, renal, or neuromuscular disease); severe obstetric complications related to the birth index (for example, eclampsia, severe postpartum hemorrhage requiring transfusion/ICU), or neonatal complications; and those who refused to participate. Multiple gestations were allowed; parity and plurality were noted.

### 2.3. Sampling and Recruitment

Sampling—Stage 2 (Convenience Sampling of Participants): Within each of the four selected primary healthcare centers, a convenience sampling approach was used to recruit participants. All women who met the following eligibility criteria during the study period (January to March 2025) were invited to participate consecutively: (a) aged 18 years or older, (b) less than or equal to 12 months postpartum at the time of interview, and (c) attending well-baby, immunization, family planning, or postnatal clinics at the participating centers. No additional randomization or stratification was applied at the individual level due to practical constraints of routine healthcare settings (e.g., limited research personnel, variable daily attendance rates, and the need to minimize disruption to clinical services).

Trained personnel conducted face-to-face interviews using a structured, validated questionnaire (the Arabic version of the SF-36). Privacy and confidentiality were strictly maintained throughout the data collection process. All information was collected solely for the purposes of this study, and participants were informed that their responses would not affect their clinical care. Thus, our sampling strategy combined cluster randomization at the facility level (to improve geographic coverage across selected areas of Amman) with consecutive convenience sampling at the participant level to maximize feasibility and recruitment efficiency.

### 2.4. Sample Size Calculation

The sample size was calculated using the formula n = N/(1 + Ne^2^), where N = population size and e = margin of error. This formula, attributed to Yamane (1967) [24], was selected because this is an exploratory study with no prior local estimates of HRQoL variability (standard deviation) among postpartum women in Jordan. In the absence of such parameters, this formula provides a conservative, pragmatic estimate of the minimum required sample size.

The population (N) was defined as 18,557, representing the estimated annual number of postpartum visits to primary comprehensive healthcare centers in Amman (Jordanian Ministry of Health). Using a 95% confidence level and a margin of error (e) of 0.05 (5%), the formula yielded a minimum required sample size of 380 participants.

To account for potential non-response, incomplete questionnaires, or missing data (estimated at 15–20% based on similar cross-sectional studies in Jordanian healthcare settings), we aimed to oversample. A total of 472 participants were successfully recruited, exceeding the minimum requirement by 24%. The final achieved margin of error was approximately 4.4%, which is within acceptable limits for cross-sectional health research.

### 2.5. Data Collection Tool

Face-to-face interviews with the study participants using the Arabic version of the Short Form Health Survey (SF-36) to measure the HRQoL of the participants after obtaining permission to conduct Research and Development (RAND) were used to collect the data [25]. SF-36 has eight domains. The domains are measured in Likert-type scales: physical functioning, role limitations caused by physical health problems, bodily pain, general health perceptions, vitality (energy/fatigue), social functioning, role limitations caused by emotional problems, and mental health (emotional well-being). Each of the domains has a 0 to 100 [26] score range, and higher scores show better QoL. The questionnaire was piloted on a small sample (e.g., n = 20) to make it clear and appropriate to the Jordanian context in terms of culture. An additional questionnaire collected social demographic as well as obstetric information such as age, marital status, education, work, income, number of children, mode of delivery, and infant feeding.

### 2.6. Statistical Analysis

IBM SPSS (version 26, IBM Corp., Armonk, NY, USA) was used to analyze the data. Descriptive statistics, including means and standard deviations (SD) for continuous variables and frequencies/percentages (%) for categorical variables, were calculated.

*To compare mean HRQoL domain scores across categories of socio-demographic and obstetric variables (e.g., marital status, employment, income groups, number of children, mode of delivery, and feeding type), independent samples t-tests (for two-group comparisons) and one-way ANOVA (for three or more groups) were performed. Bonferroni* post hoc *tests were applied to adjust for multiple comparisons following significant ANOVA results*.

Because age was the only continuous variable in our dataset, Pearson correlation coefficients were used exclusively to examine the relationship between maternal age and HRQoL domain scores. All analyses were two-tailed, and statistical significance was set at *p* < 0.05.

## 3. Results

### 3.1. Sample Characteristics

Table 1 provides the socio-demographic and obstetric characteristics of the 472 women who took part in the study postpartum. The mean age of the research participants was 30.0 years (SD = 6.83). The unemployed (69.7%) and the married (92.2%) were the majority. In relation to socioeconomic status, virtually half (47.7%) reported that the monthly household income was less than 500 JOD. Regarding obstetric history, more than half of the women (54.9%) had fewer than three children. The most common mode of delivery of birth was a normal birth (60.8%). The most frequent among the infant nutrition methods was an interim feeding method (breast and bottle) (42.2%), followed by exclusive breastfeeding (37.7%) and exclusive bottle feeding (20.1%).

### 3.2. Health-Related Quality of Life Scores

The mean scores for the eight SF-36 health domains are presented in Table 2. The highest mean score was observed in the physical functioning domain (62.48 ± 25.19), followed by bodily pain (60.39 ± 24.38) and general health perceptions (58.82 ± 15.95). In contrast, the lowest scores were reported for role limitations due to physical health (34.59 ± 37.46) and role limitations due to emotional problems (36.37 ± 40.90).

### 3.3. Association Between HRQoL and Maternal Age

Pearson correlation was carried out to determine the relationship between age and QoL domains among postpartum women (Table 3). Results revealed that there was no significant relationship between most of the QoL domains and age (*p* > 0.05). However, two domains showed a weak negative correlation, which was statistically significant at the 0.05 level: energy/fatigue (r = −0.100, *p* = 0.030) and bodily pain (r = −0.112, *p* = 0.015).

### 3.4. Associations Between Socio-Demographic Factors and Quality of Life Domains

Mean differences in HRQoL domain scores across categories of maternal socio-demographic and obstetric variables were compared using independent *t*-tests (for binary variables) and one-way ANOVA (for variables with three or more categories), as shown in Table 4 and Table 5.

Postpartum physical functioning significantly varied by marital status (F (2,469) = 4.236, *p* = 0.015) and monthly income (F (2,469) = 7.360, *p* < 0.001). The Bonferroni post hoc tests showed that the significantly higher physical functioning among married mothers compared to divorced mothers (*p* = 0.012) and the significantly better physical functioning among the highest income group (>1000 JOD) compared to both the lowest (<500 JOD, *p* < 0.001) and the middle (500–1000 JOD, *p* = 0.025) income groups. Further results showed that the type of infant feeding was significantly associated with role limitation due to emotional problems (F (2,469) = 3.387, *p* = 0.035). Post hoc analysis revealed significantly lower limitations among mothers who practiced mixed feeding than those who practiced exclusive bottle feeding (*p* = 0.046). None of the other socio-demographic variables showed statistically significant differences in these domains (all *p* > 0.05). See Table 4 for more details.

Analysis of more domains of QoL found several significant associations with socio-demographic factors (Table 5). For emotional well-being, significant differences were found across marital status (F (2,469) = 4.396, *p* = 0.013), monthly income (F (2,469) = 5.272, *p* = 0.005), and feeding type (F (2,469) = 3.451, *p* = 0.033). Bonferroni post hoc tests showed that married mothers reported significantly higher emotional well-being than divorced mothers (*p* = 0.020), and a household income of more than 1000 JOD reported significantly higher scores than the lowest income group (*p* = 0.005). Also, mixed-feeding mothers reported significantly better emotional well-being than bottle-feeding mothers (*p* = 0.031). For social functioning, unemployed mothers were found to report significantly higher scores than their employed counterparts using an independent *t*-test (t (470) = 2.909, *p* = 0.004). For bodily pain, a significant difference was found across marital status (F (2,469) = 3.574, *p* = 0.029), where post hoc analysis revealed that divorced mothers reported significantly lower pain (better status) than married mothers (*p* = 0.043). General health perception has a significant relation with marital status, monthly income, and number of children. Post hoc test revealed that the mean score of general health perception among married respondents is significantly higher than that of those classified in the other category. The lowest mean in general health perception was found among middle-income respondents, while the highest mean was found in the highest-income group. Respondents with fewer than three children reported better general health perceptions compared to those with 3–5 and more than five children. No other variables demonstrated significant associations with these domains (see Table 5 for details).

## 4. Discussion

### 4.1. Main Findings

This cross-sectional study among 472 postpartum women who attended primary healthcare centers in Amman revealed that they rated HRQoL dimensions as having the highest mean scores for physical functioning and the lowest mean scores for role limitations due to physical and emotional problems. More specifically, their quality of life was significantly associated with several socio-demographic factors. Physical functioning, general health, and emotions were found to be better in households with a higher household income and where the respondents were married. Older age only slightly correlates with less energy and more pain in the body. Selective associations were found between employment status, infant feeding, and social functioning, as well as role limitation due to emotion.

Postpartum women differ across QoL dimensions. Physical functioning recorded the highest mean score, implying that women could still perform their normal duties despite challenges in the postpartum period. The lowest mean scores were for constraints on role performance due to emotional or personal problems and physical health, however. This finding is consistent with previous research conducted in Nepal and Ethiopia using SF-36, where physical functioning was relatively better, but lower scores were seen for role-physical and role-emotional dimensions [27,28]. Similarly, a cross-sectional study among 321 postpartum women in North-East Romania reported the highest SF-36 scores in bodily pain (69.25 ± 28.87) and physical functioning (65.42 ± 14.78), while the lowest scores were observed in vitality (44.57 ± 20.20) and general health perceptions (48.73 ± 25.99)—a pattern closely mirroring our findings [29]. The consistency of these patterns across diverse cultural contexts (Middle East, South Asia, Eastern Europe) suggests that role limitations may represent a universal postpartum challenge rather than a culture-specific phenomenon, though the severity likely varies with social support systems and maternity policies. This suggests that contextual factors such as household workload, childcare burden, and restricted leave policies inhibit full recovery of mobility to perform roles. In this context of Jordanian society, such results would most probably be exacerbated by cultural expectations regarding motherhood and other domestic responsibilities imposed on women who are mainly primary caregivers, even when they themselves need a recovery. The great value of maternal duty may cause women to forsake their own physical and emotional needs;; thus, when they cannot fulfill these compounded expectations, a perception of severe role limitation.

Two aspects of QoL showed significant negative correlations with age: energy/fatigue (r = −0.100, *p* = 0.030) and bodily pain (r = −0.112, *p* = 0.015). These findings indicate that older postpartum women reported significantly lower energy levels and greater bodily pain compared to younger women. However, age was not significantly associated with physical functioning (r = −0.007, *p* = 0.871), suggesting that older women in our sample did not perceive themselves as having more limitations in performing daily physical activities despite reporting more pain and fatigue. The negative correlation between age and energy/fatigue is consistent with the results of an Iranian study that noted strong correlations among women below 30 years old between energy, social functioning, and bodily pain [30]. However, our finding regarding bodily pain differs from a Brazilian study, which found no correlation between age and QoL scores for postpartum women [31]. These discrepancies may be explained by cultural differences in pain reporting, variations in social support structures, or differences in the demographic composition of the samples. Perhaps younger women have better access to support networks, as well as faster recovery times and more tolerance for physical discomfort. In addition, older women may have more psychological and physical stress that could add to pain as well as tiredness. A longitudinal study of over 1000 women confirmed that fatigue and depressive symptoms, while correlated, are distinct psychological constructs that should be assessed separately [32]. Using confirmatory factor analysis on the SF-36 Vitality subscale and Edinburgh Postnatal Depression Scale data at 6 months and 4 years postpartum, Giallo et al. demonstrated that a two-factor model (fatigue and depression as separate but related constructs) provided a better fit than a one-factor model at both time points [32]. This finding has important implications for our study: the lower energy/fatigue scores observed among older postpartum women may reflect genuine age-related differences in physical recovery rather than underlying depression, and interventions targeting fatigue specifically (e.g., sleep hygiene, task delegation, energy conservation strategies) may be more appropriate than mental health interventions for this subgroup.

Another major finding was the relationship between employment status and social functioning. Unemployed women scored better. This is different from results in Western contexts, where employment usually correlates with good well-being, but is very understandable within this specific socio-cultural setting of Jordan. For the majority of Jordanian women, working outside the home does not seem to be an avenue for empowerment. Instead, it is a financial compulsion that creates a “double burden” situation—having full responsibilities for domestic/childcare work in addition to outside work—thus resulting in role overload and social strain [19]. A recent analysis of postpartum quality of life determinants identified ‘work-breastfeeding time conflicts’ as a primary mechanism linking employment to reduced HRQoL, noting that employed mothers face ‘time management conflicts’ between professional obligations and infant feeding demands [33]. These conflicts are exacerbated when maternity leave policies are insufficient—a study from China found that maternity leave of 4 months or less was associated with significantly lower postpartum well-being compared to leave of 6 months or more (OR = 4.85, 95% CI: 1.12 to 21.06, *p* = 0.035) [34]. The social functioning domain describes or assesses regular social activities’ functionality based on health conditions. Unemployed mothers may have flexible time to perform these extended family roles and obligations, which are highly valued in Jordanian culture, providing important support after childbirth [22]. One study from Iran explained that there is no significant relationship between occupational position and social functioning [30]. Another study from Nepal described that working women have better scores on the overall aspect of QoL [27].

It found a significant association between marital status and four domains: physical functioning, emotional well-being, bodily pain, and general health perception. This means that postnatal women might receive more avenues for activities and rest that are accompanied by social support from husbands as well as household members. Married women also reported more physical discomfort and difficulties with their physical functioning. Perhaps this is simply because they have more household chores to perform. The critical role of partner support is further quantified in a recent study published in BMJ Open, which found that total partner support was a strong independent associated factor of postpartum HRQoL (β = 0.55, 95% CI: 0.29 to 0.79, *p* < 0.001) [35]. This same study demonstrated that current breastfeeding was associated with a 72-point increase in HRQoL scores (β = 72.26, 95% CI: 60.42 to 84.10, *p* < 0.001), underscoring the interconnectedness of partner support, infant feeding, and maternal well-being. Marital satisfaction was one of the strongest associated factors of better postpartum QoL in a Korean study since a stable partnership could offer emotional support, practical help in caring for an infant, and shared housework responsibilities [36]. Previous research has identified relationship quality, spousal support, and shared caregiving as protective factors for maternal mood and HRQoL; marital strain and being separated or single were associated with increased risk for depressive symptoms and functional impairment [37,38]. Divorced women reported lower physical functioning and emotional well-being relative to their married counterparts, highlighting the value of social support in the postpartum period. Meanwhile, though unexpected, reporting less bodily pain by divorced women may be due to role redistribution and reporting of pain differently between groups or residual confounding—namely parity and prevalence of cesarean—that should be interpreted with caution and further adjusted for in multivariable models.

In this study, physical functioning, emotional well-being, and general health perception were related to the income level. Although higher scores of QoL are usually associated with high income, other studies did not find such a correlation [30,31]. However, lower postpartum QoL was reported among women with low income, as supported by the study performed by Rukiye Dikmen et al. [13]. Such differences may be attributed to the fact that many other factors, except income, such as family dynamics, psychological state, and availability of medical care for the population, determine the QoL. Also, women may underreport their financial status, which creates a variation in the relationship between income and quality of life from study to study.

The results logically confirm that the number of children significantly influences the general health perception score. With all physical energy drained from a woman due to continuous pregnancies, childbirths, and childrearing, it logically follows that her general health perception would be low; thus, vitality is lessened [15]. Successive children increase the domestic workload and stretch financial and emotional resources to more explicit negative impacts on general health perception by women. Pratiksha Chapagain et al. found no effects of the number of living children on QoL scores [27]. Prior experience may help women with more children manage postpartum issues and shape their health perceptions.

Findings in our study showed that there is no association between mode of delivery and the eight QoL domains. Sadat et al. and Pratiksha Chapagain et al. found that cesarean section deliveries were associated with poorer HRQoL scores [27,39]. In contrast, previous studies reported that women with vaginal deliveries were more likely to have better HRQoL scores [17,40].

Also, the feeding method had a significant relationship with role limitations due to personal or emotional problems, as well as emotional well-being. Our finding that mixed feeding was associated with better emotional well-being compared to exclusive bottle feeding aligns with recent evidence highlighting the psychosocial benefits of breastfeeding. A large multi-country analysis identified that mixed feeding provides mothers with greater ‘autonomy in feeding adjustments’ and ‘control over feeding adjustments’ compared to exclusive bottle feeding, which may enhance maternal self-efficacy and reduce role limitation [33]. Furthermore, a Chinese study found that formula or mixed feeding was associated with a significantly higher risk of postpartum emotional problems compared to exclusive breastfeeding (OR = 2.41, 95% CI: 1.11 to 5.24, *p* = 0.026) [34]. The protective effect of any breastfeeding (exclusive or mixed) may be mediated by oxytocin release during skin-to-skin contact, which has well-documented positive effects on mood, emotional attachment, and stress reduction. However, it is important to note that our study found no significant difference between exclusive breastfeeding and mixed feeding groups, suggesting that any degree of breastfeeding may confer emotional benefits relative to exclusive bottle feeding.

### 4.2. Strengths and Limitations of the Study

This study is the first in Jordan to focus on QoL dimensions, filling a gap in postpartum healthcare research. The use of the SF-36 tool across various healthcare facilities in Amman provides valuable data. This promotes maternal health research in Jordan while observing ethical considerations and prioritizing the health of postpartum women. Additionally, apart from maternal age, all other socio-demographic and obstetric variables were categorical, which limited our ability to examine dose–response relationships or correlations with continuous measures such as parity as a continuous variable or number of prenatal visits.

However, the following limitations should be considered when interpreting our findings: First, this study employed a cross-sectional design; therefore, all findings should be interpreted as associations rather than definitive causal relationships. Temporal precedence cannot be established, and reverse causality cannot be ruled out. For example, while we found that lower income was associated with worse HRQoL, we cannot determine whether low income causes poor HRQoL or whether poor HRQoL leads to reduced earning capacity. Second, a cross-sectional design only captures a single moment in the postpartum period (up to 12 months) and thus does not allow time-related changes to be traced appropriately. HRQoL may fluctuate significantly across the postpartum trajectory, and our findings represent a snapshot rather than a longitudinal picture. Third, recruitment was limited to primary healthcare centers in Amman city; hence, results cannot be generalized to rural populations, refugee populations (including Syrian and Palestinian refugees), or other governorates within Jordan. Fourth, possible selection bias may have been introduced through the convenience sampling approach at the participant level. Women who were experiencing severe postpartum complications or depression may have been less likely to attend the clinics and therefore less likely to be recruited. Fifth, recall bias may be present due to self-reported data on the SF-36 survey, particularly for items asking about health status over the preceding four weeks. Sixth, as noted in the statistical analysis section, multiple linear regression was not performed due to violations of assumptions; therefore, we could not adjust for potential confounding variables. Future studies with larger sample sizes should employ multivariable regression to identify independent associated factors.

Additionally, although the study included women up to 12 months postpartum, the exact time since childbirth was not analyzed as a separate variable. HRQoL may differ considerably between early and late postpartum stages; therefore, temporal variations in postpartum recovery could not be assessed in the present study. Future research should stratify participants according to postpartum duration or employ longitudinal designs to better understand HRQoL trajectories over time.

Despite these limitations, this study provides crucial baseline data on postpartum HRQoL in Jordan and identifies key areas for intervention and policy development.

### 4.3. Interpretation

The findings of this study highlight how postpartum HRQoL in Jordan is shaped by a complex interplay of socio-cultural and economic factors. The consistent association between low income and reduced HRQoL underscores the influence of financial stability on access to healthcare, nutrition, and psychosocial well-being—findings that align with evidence from other middle-income countries where socioeconomic inequalities exacerbate maternal health disparities [14,15]. Moreover, the marked differences in HRQoL by marital status and employment point to the dual burden many Jordanian women face between domestic and occupational responsibilities, echoing global findings that unpaid care work intensifies stress and limits recovery after childbirth [36,38]. These results suggest that interventions aimed at improving postpartum well-being should move beyond medical support to include social and economic empowerment initiatives. Integrating culturally sensitive counseling, family-inclusive education, and community-based support systems could mitigate the emotional and physical limitations identified in this study. Ultimately, the study’s findings reinforce that maternal health programs in Jordan must address structural determinants of health—income inequality, gender roles, and social support networks—to sustainably enhance postpartum HRQoL.

## 5. Conclusions

This study identified lower HRQoL scores across several domains among postpartum women attending selected primary healthcare centers in Amman, Jordan, with role limitations as the most affected dimension. Influencing factors are multidimensional, spanning socio-demographic and obstetric contexts. These results bear great meaning for clinical practice and public health policy consideration.

The findings of this study suggest the potential value of moving beyond the traditional forty-day, biologically focused model of postpartum care in Jordan. Routine HRQoL screening may help identify women at risk of reduced postpartum well-being. Multidimensional interventions could be considered to support postpartum well-being, including: psychoeducational couple interventions to enhance marital support while managing role expectations; financial support programs or community-based interventions for low-income families; community health worker programs for practical support and social connection, especially for women with limited family support; and flexible, supportive breastfeeding counseling that reduces maternal anxiety.

In light of our finding that unemployed women reported significantly better social functioning than employed women—suggesting that employment in Jordan’s current context may create a ‘double burden’ rather than empowerment—the findings may have implications for workplace support policies for postpartum mothers, including the following possible measures:

- Workplace childcare facilities: On-site or near-site affordable childcare to reduce the logistical and emotional burden of arranging infant care while working.
- Flexible working hours: Adjusted start and end times, compressed workweeks, or reduced schedules during the first six months postpartum to allow women to manage fatigue, breastfeeding, and childcare responsibilities.
- Extended paid maternity leave: Current Jordanian labor law provides 10 weeks of paid maternity leave. Extending this to at least 4–6 months (consistent with WHO recommendations) could allow for adequate physical recovery and establishment of breastfeeding before returning to work.
- Protected breastfeeding breaks: Guaranteed paid breaks for breastfeeding or pumping, along with private, clean spaces for expressing milk, as recommended by the International Labor Organization.
- Return-to-work support programs: Structured re-entry programs that include gradual return-to-work schedules, peer support groups for working mothers, and supervisor training on postpartum mental health.

Such policies could potentially reduce role strain among employed postpartum women and may contribute to improved HRQoL outcomes. Future studies should utilize a longitudinal design to observe HRQoL trajectories and evaluate the effectiveness of such targeted interventions in enhancing the well-being of Jordanian mothers.

## Figures and Tables

**Table 1:** Socio-demographic and Obstetric Characteristics (N = 472).

Variables	Frequency (%)
**Age, mean (SD)**	30 (6.83) range 18–44
**Employment status**	
Unemployed	329 (69.7)
Employed	143 (30.3)
**Marital status**	
Married	435 (92.2)
Divorced	16 (3.4)
Others	21 (4.4)
**Monthly Income (JOD)**	
<500	225 (47.7)
500–1000	176 (37.3)
>1000	71 (15.0)
**Children numbers**	
<3 children	259 (54.9)
3–5 children	196 (41.5)
>5 children	17 (3.6)
**Mode of Delivery**	
Normal vaginal	287 (60.8)
Cesarean section	185 (39.2)
**Type of feeding**	
Breast feeding	178 (37.7)
Bottle	95 (20.1)
Mixed	199 (42.2)

**Table 2:** Mean Score of SF-36 HRQoL of postpartum women.

SF-36 Health Domain	Mean	SD	95% CI
Physical functioning	62.48	25.19	60.20–64.76
Role limitations due to physical health problems	34.59	37.46	31.20–37.98
Role limitations due to personal or emotional problems	36.37	40.90	32.67–40.70
Energy/fatigue	44.75	16.97	43.21–46.28
Emotional well-being	49.29	17.69	47.69–50.89
Social functioning	56.97	22.85	54.90–59.03
Bodily pain	60.39	24.38	58.18–62.59
General health perceptions	58.82	15.95	57.38–60.27

Note: SF-36 = 36-Item Short Form Survey. Scores range from 0 to 100, with higher scores showing better quality of life. SD: Standard Deviation.

**Table 3:** Relationship between HRQoL and age among postpartum women.

Quality of Life Among Postpartum Women	Pearson Correlation (r)	Sig. (2-Tailed)
Physical functioning	−0.007	0.871
Role limitations due to physical health problems	−0.041	0.376
Role limitations due to personal or emotional problems	−0.042	0.361
Energy/fatigue	−0.100	0.030
Emotional well-being	−0.030	0.512
Social functioning	−0.032	0.484
Bodily pain	−0.112	0.015
General health perceptions	−0.020	0.663

**Table 4:** Comparison of mean HRQoL domain scores (physical functioning, role limitations due to physical health problems, role limitations due to emotional problems, and energy/fatigue) across categories of socio-demographic and obstetric characteristics among postpartum women (N = 472).

Variables	Physical Functioning			Role Limitations Due to Physical Health Problems			Role Limitations Due to Personal or Emotional Problems			Energy/Fatigue		
	Mean ± SD	Test-Value	*p*-Value	Mean ± SD	Test-Value	*p*-Value	Mean ± SD	Test-Value	*p*-Value	Mean ± SD	Test-Value	*p*-Value
**Employment status**		1.31	0.191		0.86	0.391		1.54	0.123		1.35	0.177
Unemployed	63.48 ± 25.60			35.56 ± 28.11			50.12 ± 18.47			45.44 ± 17.37		
Employed	60.17 ± 24.15			32.34 ± 25.95			47.38 ± 15.66			43.15 ± 15.97		
**Marital status**		4.24	0.015		1.28	0.280		2.58	0.077		1.66	0.191
Married	63.18 ± 24.98 ^a^			34.94 ± 37.42			36.86 ± 41.02			44.71 ± 16.85		
Divorced	44.69 ± 26.11 ^b^			20.31 ± 20.31			14.58 ± 29.74			50.94 ± 9.70		
Others	61.43 ± 25.01			38.10 ± 40.01			42.86 ± 42.35			40.71 ± 22.38		
**Monthly Income (JOD)**		7.36	>0.001		0.33	0.718		0.80	0.448		0.35	0.770
<500	59.16 ± 24.72 ^a^			34.56 ± 37.99			34.37 ± 40.26			44.62 ± 15.48		
500–1000	62.84 ± 25.58 ^a^			33.38 ± 36.61			36.93 ± 41.01			45.43 ± 17.76		
>1000	72.11 ± 23.45 ^b^			37.68 ± 38.24			41.31 ± 42.71			43.45 ± 19.49		
**Children numbers**		0.003	0.997		1.25	0.286		0.24	0.785		0.25	0.783
<3 children	62.45 ± 26.85			33.69 ± 37.12			36.04 ± 41.68			44.58 ± 17.28		
3–5 children	62.55 ± 22.72			34.57 ± 37.22			36.22 ± 39.33			45.18 ± 16.61		
>5 children	62.06 ± 27.73			48.53 ± 44.61			43.14 ± 48.25			42.35 ± 17.15		
**Mode of Delivery**		1.25	0.212		0.19	0.853		068	0.497		0.91	0.366
Normal vaginal	63.64 ± 24.55			34.84 ± 37.76			37.40 ± 41.38			45.31 ± 16.66		
Cesarean section	60.68 ± 26.12			34.19 ± 37.09			34.77 ± 40.20			43.86 ± 17.46		
**Type of feeding**		1.04	0.353		0.60	0.551		3.39	0.035		0.79	0.457
Breast feeding	61.69 ± 24.62			36.24 ± 38.97			38.58 ± 41.44			45.70 ± 16.56		
Bottle	60.11 ± 27.46			31.05 ± 34.73			26.67 ± 41.59			43.00 ± 16.54		
Mixed	64.32 ± 27.46			34.80 ± 37.41			39.03 ± 41.59			44.72 ± 17.55		

Different superscripts indicate a statistically significant mean difference.

**Table 5:** Comparison of mean HRQoL domain scores (emotional well-being, social functioning, bodily pain, and general health perceptions) across categories of socio-demographic and obstetric characteristics among postpartum women (N = 472).

Variables	Emotional Well-Being			Social Functioning			Bodily Pain			General Health Perceptions		
	Mean ± SD	Test-Value	*p*-Value	Mean ± SD	Test-Value	*p*-Value	Mean ± SD	Test-Value	*p*-Value	Mean ± SD	Test-Value	*p*-Value
**Employment status**		1.54	0.123		2.91	0.004		0.02	0.985		0.59	0.559
Unemployed	50.12 ± 18.47			58.97 ± 23.76			60.37 ± 24.85			58.54 ± 16.15		
Employed	47.38 ± 15.66			52.36 ± 19.96			60.42 ± 23.35			59.48 ± 15.52		
**Marital status**		4.40	0.013		2.31	0.100		3.57	0.029		3.58	0.029
Married	49.98 ± 17.73 ^a^			57.61 ± 22.59			59.52 ± 24.50 ^a^			59.34 ± 16.07 ^a^		
Divorced	39.50 ± 10.11 ^b^			50.78 ± 18.52			72.03 ± 21.10 ^b^			55.94 ± 6.12		
Others	42.48 ± 18.52			48.21 ± 29.12			69.40 ± 20.49			50.24 ± 16.24 ^b^		
**Monthly Income (JOD)**		5.27	0.005		1.63	0.196		0.33	0.722		5.78	0.003
<500	47.06 ± 16.71 ^a^			55.94 ± 20.36			59.48 ± 24.44			57.22 ± 14.88		
500–1000	49.98 ± 18.02			56.46 ± 24.49			60.98 ± 24.08			58.58 ± 16.53 ^a^		
>1000	54.65 ± 18.86 ^b^			61.44 ± 25.77			61.80 ± 25.13			64.51 ± 16.67 ^b^		
**Children numbers**		0.63	0.534		0.60	0.551		0.38	0.685		4.56	0.011
<3 children	49.98 ± 19.34			57.87 ± 23.14			61.21 ± 25.04			60.62 ± 16.87		
3–5 children	48.67 ± 15.48			56.12 ± 22.31			59.55 ± 23.39			57.07 ± 14.47		
>5 children	45.88 ± 15.50			52.94 ± 25.21			57.50 ± 26.26			51.76 ± 14.25		
**Mode of Delivery**		1.03	0.303		0.46	0.646		1.72	0.083		0.87	0.384
Normal vaginal	48.61 ± 17.09			56.58 ± 22.19			61.93 ± 23.51			59.34 ± 14.84		
Cesarean section	50.34 ± 18.60			57.57 ± 23.89			57.99 ± 25.55			58.03 ± 17.53		
**Type of feeding**		3.45	0.033		1.20	0.303		0.12	0.886		1.39	0.251
Breast feeding	49.82 ± 17.40			57.02 ± 22.74			61.01 ± 24.50			58.03 ± 14.93		
Bottle	45.14 ± 18.05 ^a^			53.95 ± 22.65			59.53 ± 25.63			57.37 ± 16.35		
Mixed	50.79 ± 17.54 ^b^			58.35 ± 23.03			60.24 ± 23.75			60.23 ± 16.59		

Different superscripts indicate a statistically significant mean difference.

## Data Availability

Data presented in this study are included in the article; further inquiries can be directed to the corresponding author.

## Abbreviations

The following abbreviations are used in this manuscript:

HRQoL	Health-related Quality of Life
SF-36	36-Item Short Form Survey
QoL	quality of life
RAND	Research and Development
SD	Standard Deviations
